# Innovative Application of a Rigidizing Overtube in Endoscopic Ultrasound–Guided Biopsy for Sampling of a Cecal Submucosal Lesion

**DOI:** 10.14309/crj.0000000000001722

**Published:** 2025-05-30

**Authors:** Anas Zaher, Nicholas G. Brown

**Affiliations:** 1 Department of Internal Medicine, New York-Presbyterian Brooklyn Methodist Hospital, Brooklyn, NY; 2 Department of Gastroenterology and Hepatology, New York-Presbyterian Brooklyn Methodist Hospital, Brooklyn, NY

**Keywords:** rigidizing overtube, subepithelial lesion, endoscopic ultrasound

## Abstract

Subepithelial lesions in the gastrointestinal tract present diagnostic challenges, particularly in difficult-to-access regions like the cecum. This case report describes a 71-year-old woman with a submucosal lesion adjacent to the appendix, where a rigidizing overtube was used to stabilize the colonoscope for an endoscopic ultrasound–guided fine-needle biopsy. The procedure was successful, obtaining a benign diagnosis without the need for surgery. This case highlights the utility of rigidizing overtube technology in enhancing the accuracy and safety of endoscopic interventions for subepithelial lesions, particularly in anatomically challenging locations like the cecum.

## INTRODUCTION

Subepithelial lesions (SEL) of the gastrointestinal (GI) tract are often detected incidentally and present unique diagnostic challenges due to their submucosal location.^1^ Endoscopic ultrasound (EUS) has become an essential tool in the assessment and biopsy of these lesions, offering precise visualization and tissue acquisition. Traditionally, in challenging locations such as the cecum, the limited accessibility and stability required for accurate biopsy often necessitated partial surgical resection of the colon. We present a 71-year-old woman who was found to have a submucosal lesion adjacent to the appendix. Using the rigidizing overtube, we successfully performed an EUS-guided fine-needle biopsy (FNB) without the need for surgery. This case demonstrates the potential of rigidizing overtube technology to enable minimally invasive management of complex colonic lesions.

## CASE REPORT

A 71-year-old woman with a medical history of hypertension presented to the gastroenterology clinic with persistent abdominal discomfort. For the past 2 years, she had experienced an intermittent, shock-like sensation in the right upper quadrant, occasionally extending to the left upper quadrant. This sensation did not vary with changes in bowel habits or food intake. Her social history was notable for being a nonsmoker and occasional alcohol consumption. She denied any family history of GI malignancy.

Her abdominal exam was unremarkable. Initial workup included a urea breath test for *Helicobacter pylori*, which returned positive; the patient was subsequently started on appropriate therapy. Further evaluation with colonoscopy revealed a submucosal lesion near the appendix. Tunneled biopsy was performed with cold forceps. However, the histology results showed no malignancy. To better characterize this finding, a computed tomography scan of the abdomen and pelvis with contrast was obtained, which confirmed the presence of a 1.1-cm submucosal lesion adjacent to the appendix orifice.

A multidisciplinary team reviewed the case and recommended further assessment using a through-the-scope probe. Although surgical resection vs endoscopic resection was discussed, the patient expressed a preference to avoid invasive management for the time being.

**Figure 1.**
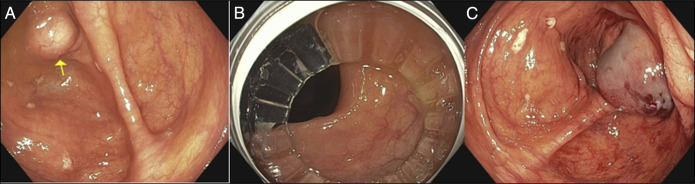
Endoscopic imaging and procedural views: (A) endoscopic view of the subepithelial lesion in the cecum. (B) Colonoscope inserted through the rigidizing overtube. (C) Post–fine-needle aspiration image showing the biopsy site after a single-pass FNB with a 22-gauge needle.

During colonoscopy, the entire colon was examined and appeared otherwise normal. To facilitate precise imaging and biopsy, a rigidizing overtube was deployed onto the colonoscope and advanced to the cecum, where it was left in place while the colonoscope was exchanged for a linear echoendoscope (Figure 1). The EUS portion of the examination confirmed the presence of a round, intramural (subepithelial) lesion in the cecum, adjacent to the appendix. The lesion appeared hypoechoic, with its origin located within the deep mucosa. It measured approximately 11 mm × 9 mm, with well-defined endosonographic borders.

A fine-needle biopsy was performed using a 22-gauge biopsy needle via a transcolonic approach, yielding a core tissue sample. Cytological examination of the specimen confirmed benign pathology. The procedure was successful, and the patient tolerated it well, leaving without complications.

## DISCUSSION

The diagnostic approach to SEL in the GI tract has evolved significantly with advancements in endoscopic tools and techniques. Historically, lesions in difficult-to-access regions, such as the cecum, often required invasive surgical procedures for definitive diagnosis due to limited maneuverability and stability within the colon. The introduction of devices like the rigidizing overtube has revolutionized this field, enabling safer and more effective endoscopic interventions even in anatomically challenging areas.^2^

**Figure 2.**
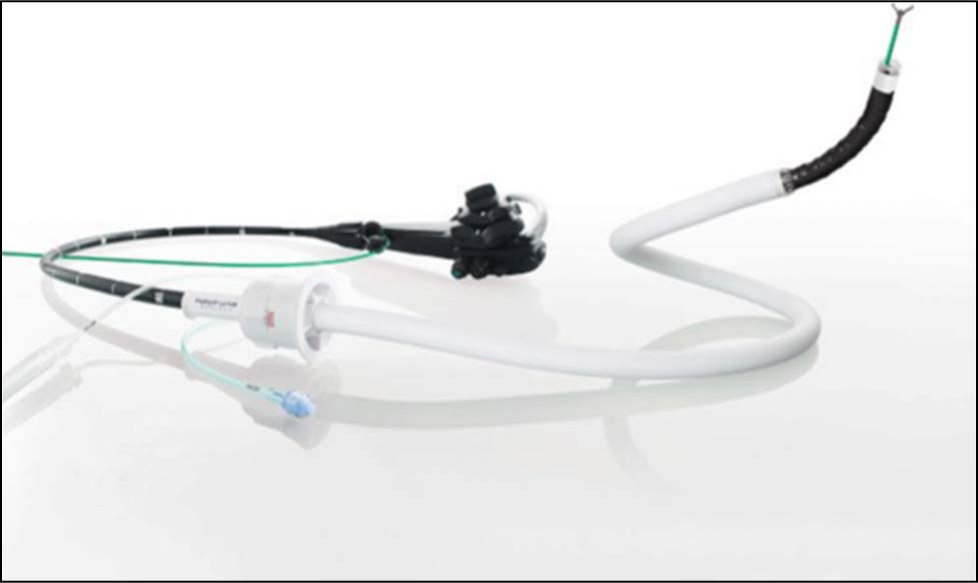
Rigidizing overtube, single-use flexible overtube.

For submucosal lesions, the American Gastroenterological Association recommends using EUS with fine needle aspiration or fine needle biopsy (FNB) to obtain tissue samples and determine the nature of the lesions. However, assessing SEL in the right colon, including the cecum, poses unique challenges due to anatomical positioning and potential loop formation, making precise needle placement difficult.^2^ The rigidizing overtube (Figure 2) has traditionally been applied in procedures where loop formation is a significant issue, including colonoscopy, enteroscopy, and endoscopic retrograde cholangiopancreatography in patients with altered anatomy. Initially flexible for navigating GI tract bends, the rigidizing overtube stiffens upon vacuum application, creating a stable conduit that maintains the endoscope's position without looping ^3^. This mechanism improves procedural efficiency and safety by reducing repeated scope passes and mitigating procedural fatigue from prolonged manipulation.^4–6^

The safety of the rigidizing overtube has been demonstrated in clinical use. Jawaid et al.^7^ described its application in 97 patients without any device-related adverse events, supporting its role as safe adjunct in complex endoscopic procedures.

According to our knowledge, this is the first reported case in the literature utilizing a rigidizing overtube for FNB of a cecal lesion with EUS guidance. In this case, the use of the rigidizing overtube device facilitated a successful, single-pass FNB with optimal tissue acquisition, allowing for a definitive benign diagnosis. Such advancements underscore the potential of modern endoscopic tools in expanding access to regions of the GI tract previously deemed surgically challenging, offering patients an effective and less invasive pathway to accurate diagnosis and management.

The study's strength lies in its novel use of the rigidizing overtube for EUS-guided FNB of a cecal lesion. However, as a single case report, its findings lack generalizability. Larger studies are needed to confirm its benefits.

## DISCLOSURES

Author contributions: A. Zaher: manuscript drafting, chart review and data abstraction, manuscript revision, guarantor of manuscript. NG Brown: manuscript revision. Anas Zaher is the article guarantor.

Financial disclosure: None to report.

Informed consent was obtained for this case report.
